# Serous carcinoma arising from uterine adenomyosis/adenomyotic cyst of the cervical stump: a report of 3 cases

**DOI:** 10.1186/s13000-016-0496-0

**Published:** 2016-06-04

**Authors:** Bingjian Lu, Qin Chen, Xiaofei Zhang, Lili Cheng

**Affiliations:** Department of Surgical Pathology, the Affiliated Women’s Hospital, School of Medicine Zhejiang University, Hangzhou, People’s Republic of China 310006; Department of Gynecologic Oncology, Women’s Hospital, School of Medicine, Zhejiang University, Hangzhou, Zhejiang Province People’s Republic of China

**Keywords:** Endometrial serous carcinoma, Serous intraepithelial carcinoma, Endometrial glandular dysplasia, Adenomyosis, Adenomyotic cyst, Immunohistochemistry

## Abstract

**Background:**

Serous carcinoma arising in adenomyosis and adenomyotic cyst is very rare. Only 3 serous carcinomas and 5 serous endometrial intraepithelial carcinomas (EIC) have been reported to date.

**Methods:**

We reviewed the clinicopathological features of 2 serous carcinoma in uterine adenomyosis and 1 serous EIC in adenomyotic cyst of the cervical stump.

**Results:**

Case 1 had an endometrial serous carcinoma in the uterine myometrium and the left ovary. A minor component of benign endometrial glands with minimal endometrial stroma was found in the uterine mass and the surrounding myometrium. Case 2 showed 3 small foci of serous carcinoma, serous EIC and endometrial glandular dysplasia (EmGD) in the adenomyosis. Scanty serous carcinoma was present in the endometrium without evidence of myometrial invasion. The eutopic endometrium in both case 1 and 2 had no evidence of neoplastic changes after complete examination. Case 3 had 3 microscopic serous EICs in the adenomyotic cysts of the cervical stump. One EIC lesion coexisted with EmGD. No cancer was found in the endocervical tube although the preoperative endocervical biopsy showed a poorly differentiated endometrioid carcinoma. Immunohistochemistry demonstrated that serous carcinoma in case 1 and EIC in all 3 cases showed a characteristic pattern of p53 and p16 over expression, high Ki67 index, and lack of WT1, ER and PR staining. EmGD in case 1 and 3 had a similar staining pattern except a lower Ki67 index and the presence of ER expression.

**Conclusions:**

We believe that this case series may expand our recognition on serous carcinoma arising in uterine adenomyosis/adenomyotic cyst including extra-uterine spread and the potential synchronous growth of carcinomas in eutopic endometrium.

## Background

Carcinomatous changes from the ectopic endometrial glands in endometriosis have been reported in many studies [1–4]. Most cases occur in patients with ovarian endometriosis. Malignant transformation from uterine adenomyosis is very unusual. Less than 50 cases have been reported currently although the first case was reported in 1897. The predominant histotypes of these cases are endometrioid carcinoma and clear cell carcinoma [4–6]. Serous carcinoma arising in uterine adenomyosis is extremely rare. Only 3 reports containing 3 serous carcinomas and 5 serous endometrial intraepithelial carcinomas (EIC) have been reported to date [7–10]. In this study, we present 2 serous carcinomas in uterine adenomyosis and 1 serous EIC in adenomyotic cyst of the cervical stump.

## Methods

The 3 cases reported here were retrieved from the archives of the Department of Surgical Pathology, the Affiliated Women’s Hospital School of Medicine Zhejiang University, China, in the last 5 years. Patients 1 and 2 were recent cases. We obtained clinical details and follow-up data from hospital medical records. This study was approved by the Institutional Research Ethics Committee of the Affiliated Women’s Hospital School of Medicine Zhejiang University, China. The patients were well informed and consent with this study. Archival routine hematoxylin and eosin stained slides were assessed by the authors.

Additional 4 μm sections were cut for immunohistochemistry. The sources and dilutions of a group of antibodies are detailed in Table 1. A two-step En Vision immunostaining procedure (DAKO, Carpentaria, CA, USA) was performed according to the manufacturer’s protocols. The percentage of positive cells was scored as follows: − for no immunoreactivity; focally + for 1 % to 5 %; + for 6 % to 25 %; ++ for 26 % to 50 %; +++ for 51 % to 75 %; ++++ for 76 % to 100 %. Over expression of p16 and p53 were defined at least “+++”.

**Table 1:** Antibody clones, sources, and dilutions

Antibody	Clones	Dilutions	Sources
ER	1D5	1:300	Thermo
PR	1A6	1:500	Thermo
CK7	OV-TL	1:100	Thermo
CK20	KS20.8	1:100	Leica
PAX8	ZR1	1:50	Invitrogen
WT1	6 F-H2	1:150	Zeta
CA125	EPR1020(2)	1:50	Zeta
p53	DO-7	1:600	Thermo
CD10	56C6	1:100	Leica
D2-40	D2-40	1:400	Thermo
p16	16P04/JC2	1:100	Zeta
PTEN	17A	1:50	Zymed
β-catenin	E247	1:400	Zymed
HNF1β	C-20	1:200	Santa Cruz
Ki67	MIB-1	1:400	DAKO

## Results

### Clinical findings

Table 2 summarizes the major clinical features of all 3 cases.

**Table 2:** Clinical findings of the lesions

	Case 1	Case 2	Case 3
Age (yr)	64	55	55
History of pregnancy	G2P2	G1P1	G2P1
History of cancer	N	N	N
Familial history of cancer	N	N	Her father died of lung cancer.
Clinical presentation	Left low abdominal pain	Postmenopausal vagina bleeding	Vaginal bleeding
Serum tumor biomarkers	Normal CA125, CA199 & CA153	CA199: 89.8 IU/mL; Normal CA125	ND
Imaging findings	Endometrium 0.2 cm; A mass in the left parametrium; A cyst in the left adnexus	Endometrium 0.22 cm; Uterine leiomyomas	A mass in the cervical stump and a cyst in the left ovary
Treatment	TAHBSO + complete staging surgery	TAHBSO + complete staging surgery	Radical resection of the cervical stump + BSO + complete staging surgery
Postoperative chemotherapy	Y	Y	N
Status of follow-up (time)	Recent case	Recent case	Alive with no evidence of cancer (44 months)

*Abbreviations*: *N* no, *Y* yes, *ND* not done, *BSO* bilateral salpingo-oophorectmy, *TAHBSO* total abdominal hysterectomy and bilateral salpingo-oophorectmy

#### Case 1

A 64-year-old Chinese woman, gravida 2, para 2, complained of left low abdominal pain for about 6 months. Her last menses was more than 10 years ago. She denied history of hormone replacement therapy. She had a history of diabetes mellitus with the treatment of Diamicron and Metformin Hydrochloride for more than 10 years. She denied familial history of cancer. Pelvic examination revealed a slightly enlarged and symmetrical uterus. A pelvic sonogram showed that the endometrium strip measured 0.2 mm. An irregular mass of 2.5 × 2.6 × 2.6 cm was present in the left parametrium. A cystic lesion of 4.0 × 2.1 × 3.2 cm was found in the left adnexus. It was adjacent to the mass in the left parametirum. The serum levels of CA125, CA 199 and CA153 were normal. The clinical impression of this case was “adenomyosis and possible left fallopian tube-ovarian cyst”. Total abdominal hysterectomy and bilateral salpingo-oophorectmy (TAHBSO) was initially performed. A 4.0-cm cystic lesion was found in the left adnexus. The cystic lesion enveloped the left ovary and fallopian tube, and adhered with the uterus and intestine. However, the left ovary and fallopian tube was not clearly seen. The uterus and the right fallopian tube looked normal anatomically. After a diagnosis of “adenocarcinoma in the left adnexus” was rendered on frozen sections, a complete staging surgery including retroperitoneal lymph node dissection and omentectomy was subsequently carried out. A small focus of endometriosis from the rectal adventitia was also biopsied. The patient was given one course of TP (Taxol + Carboplatin) chemotherapy and was unremarkable for one month after surgery at present.

#### Case 2

A 55-year-old Chinese woman gravida 1, para 1, complained of postmenopausal vagina bleeding for 8 months. Uterine adenomyosis and multiple leiomyomas were found 19 years ago. She was regularly followed-up thereafter. Her serum CA199 was slightly elevated (89.8 IU/mL) 4 months ago. Serum CA125 was normal. Recent pelvic sonogram indicated that the enlarged uterus had a size similar to that of 2 weeks of gestation and the endometrium strip measured 0.22 cm. There were multiple leiomyomas in the uterus. The largest one measured 5.6 × 5.5 × 5.2 cm. Pelvic CT scan and MRI had similar findings to that of sonogram. Her medical history was significant for diabetes mellitus, primary hypertension, asthma, appendectomy and caesarean. The diagnosis of preoperative endometrial curetting was serous carcinoma. TAHBSO and complete staging surgery was performed 4 weeks after the diagnosis. One course of adjuvant chemotherapy (TP) was given postoperatively by now.

#### Case 3

A 55-year-old Chinese woman, gravida 2, para 1, complained of vaginal bleeding for 22 days. She had undergone a subtotal hysterectomy for uterine adenomyosis 16 years ago. Sonogram revealed a 4.4 × 4.4 × 3.0 cm mass in the cervical stump and a 3.3 × 3.5 × 2.8 cm cyst in the left ovary. Pelvic CT scan showed an enlarged cervical stump with a 2.5 × 1.7 × 1.5 cm mass and a 2.8 × 2.4 × 3 cm cyst in the left ovary. The pathological diagnosis of her endocervical curetting was poorly differentiated endometrioid carcinoma. Her familial history was unremarkable except that her father died of lung cancer. Radical resection of the cervical stump, bilateral salpingo-oophorectmy (BSO) and complete staging surgery were performed two weeks later. The rectum was partially removed due to the presence of endometriosis resulting in its dense adhesion with the cervix stump. She was alive with no evidence of cancer for at least 44 months.

### Pathological findings

The main pathological and immunohistochmical findings of these cases are given in Table 3 and Table 4, respectively.

**Table 3:** Pathological results of the lesions

	Case 1	Case 2	Case 3
FIGO Stage	IIIa	Ia	Ia
Gross uterine mass	-	-	-
Pathological findings in the endometrium	-	SC	EC (G2-3)
Myometrium invasion	NA	-	-
Lymphovascular tumor emboli	+	+	-
Findings in the adenomyosis/ endometriotic cyst	SC	Minimal SC; serous EIC; EmGD	Serous EIC; EmGD
Other findings	Spread to the left ovarian; Uterine leiomyoma; Endometriosis in the rectum.	Uterine leiomyoma	Glandular cyst in the cervix stump; Left ovarian endometriotic cyst; Endometriosis in the rectum.

*Abbreviations*: *SC* serous carcinoma, *EIC* serous endometrial intraepithelial carcinoma, *EmGD* endometrial glandular dysplasia, *EC* endometrioid carcinoma, *NA* not applicable

**Table 4:** Immunostaining results of the lesions^a^

Antibodies	Case 1		Case 2		Case 3	
	Adenomyosis	Left Ovary	Curetting	Adenomyosis	Curetting	Adenomyosis
ER	-	-	-	-	-	-
PR	-	-	-	-	-	-
CK7	+++	+++	ND	ND	ND	ND
CK20	-	-	ND	ND	ND	ND
PAX8	+++	+++	+++	+++	+++	+++
WT1	-	-	-	-	-	-
CA125	++	++	ND	ND	ND	ND
p53	+++	+++	+++	+++	+++	+++
CD10^a^	-	-	ND	ND	ND	ND
D2-40^a^	-	-	-	-	ND	ND
PTEN	++	++	++	++	ND	ND
β-catenin	Mem+	Mem+	Mem+	Mem+	ND	ND
p16	+++	+++	+++	+++	+++	+++
HNF1β	ND	ND	-	-	-	-
Ki67	>80 %	>80 %	>70 %	>70 %	>80 %	>80 %

*Abbreviations*: *ND* not done, *Mem* membrane staining

^a^CD10 and D2-40 was used to highlight the presence of endometrial stroma and lymphovascular invasion in the adenomyosis, respectively

#### Case 1

The uterus measured 6.5 × 3 × 3 cm. A 2.2 × 2.3 × 2.0-cm mass was found in the outer two third of the uterine myometrium. It extended from the left side of the corpus to the isthmus. The mass had a white and coarsely trabeculated cutting surface. The texture was similar to that of the surrounding myometrium. The boundary of the mass was not very clear. The myometrium surrounding the mass had foci of hemorrhage. A leiomyoma with a diameter of 0.5 cm was found in the isthmus. The endometrium was atrophic and about 0.1 cm thick. The uterine cervix was unremarkable. The specimens from the left adnexa (*n* = 4), the right fallopian tube (*n* = 2), the uterine mass, the endometrium (*n* = 9) and the cervix (*n* = 8) were all extensively sectioned for histopathological examination.

The broken specimens from the left adnexus measured 4 × 3 × 2 cm. The fallopian fimbriae and ovary was grossly unrecognizable, but the fallopian tube was partially recognizable with a diameter of 1.0 cm. The tubal wall measured 0.3-0.4 cm. Some cystic wall-like tissue had a thickness of 0.1-0.2 cm. Two pieces of grey-to-white, tender solid tissues, measured 1.5 × 1.0 × 0.8 cm and 0.8 × 0.5 × 0.5 cm, separately, were present in the broken tissues. The right fallopian tube and ovary, and omentum were grossly normal.

**Fig. 1:**
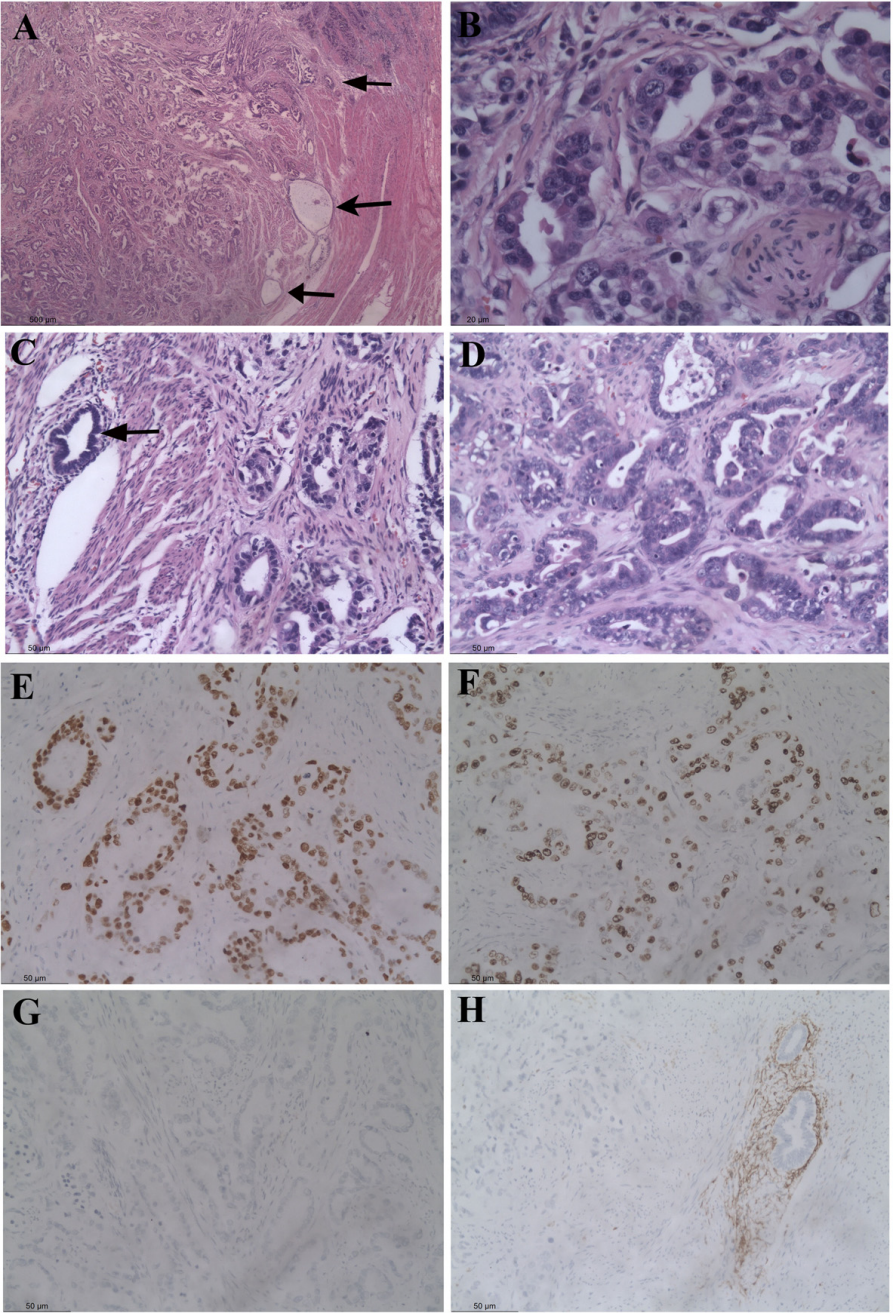
Serous carcinoma in uterine adenomyosis (case 1). The uterine mass showed neoplastic glands with marked atypia splitting smooth muscle fibers **a**, **b**. A small amount of benign endometrial glands with minimal endometrial stroma were found at the periphery of the tumor and within the tumor, implicating the presence of adenomyosis **a**, **c**: *arrows*. The identical morphological changes were seen in the left adnexus **d**. Both uterine and ovarian tumors showed an identical immunohistochemical profile including p53 over expression **e**, high Ki67 index **f**, and WT1 -ve **g**. CD10 staining highlights the presence of endometrial stroma surrounding the benign glands in the uterine tumor **h**. (Original magnifications: A*25; B*400; C-G*200)

Microscopically, the uterine mass showed neoplastic glands splitting smooth muscle fibers (Fig. 1a). It displayed an expansive growth pattern towards the surrounding myometrium. The neoplastic glands were lined by markedly atypical, cuboidal to columnar cells with hyperchromasia, conspicuous nucleoli, and irregular nuclear membrane and numerous mitotic figures (>12/10HPFs) (Fig. 1b). The luminal borders were smooth, and some had epithelial tufts. Small clusters of atypical cells surrounded by a clear space were commonly seen. However, desmoplastic stroma was inconspicuous. A small amount of benign endometrial glands with minimal endometrial stroma were present in the adjacent normal myometrium surrounding the tumor and within the tumor, implicating the presence of adenomyosis (Fig. 1a, c). Tumor emboli within the lymphovascular vessels were occasionally found within the myometrium. The atrophic endometrium showed resting changes without neoplastic evidence including cancer precursor alterations. The small nodule in the isthmus was a leiomyoma.

The solid tissues in the left adnexus showed the same morphological changes as in the uterine mass in general (Fig. 1d). However, they showed a vaguely multi-nodular growth pattern with a prominent desmoplastic reaction. There were no benign-looking endometrioid glands in the tumor. Ovarian stroma was discernable at the periphery of the lesion. The left fallopian tube and the cystic wall were in keeping with hydrosalpinx and showed no evidence of serous tubal intraepithelial carcinoma (STIC) or serous carcinoma. Biopsy from the rectal adventitia was diagnosed as endometriosis.

Tumors from the uterine and ovarian mass showed an identical immunohistochemical profile (Table 4; Fig. 1e-g). Notably, CD10 clearly demonstrated the presence of endometrial stroma surrounding the benign glands in the uterine tumor (Fig. 1h).

#### Case 2

The uterus measured 13 × 9 × 6.5 cm. There were multiple leiomyomas in the myometrium. The largest one had a diameter of 5.0 cm. Adenomyosis was diffusely found in the myometrium. It was characterized by the trabeculated cut surface and small foci of hemorrhage. No gross tumor was identified in the uterine cavity. The endometrium measured 0.1 cm and looked unremarkable. The uterus including endometrium and myometroum (*n* = 17) were completely examined. The fallopian fimbriae (*n* = 2 for each side) were also extensively investigated.

**Fig. 2:**
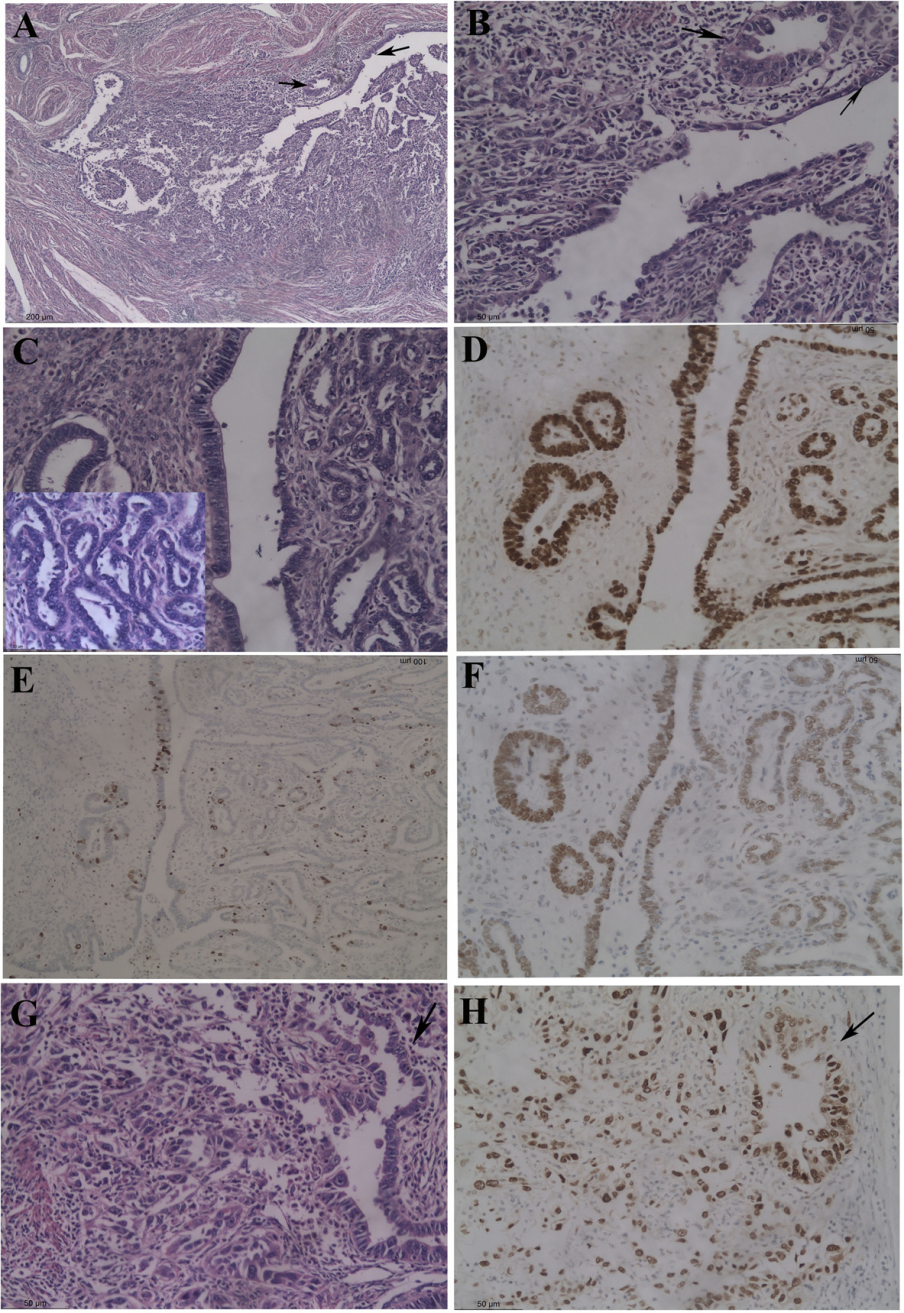
Serous EIC in uterine adenomyosis (case 2). A small focus of serous carcinoma and serous EIC (arrows) was present in the adenomyosis **a**. The neoplastic cells showed significant atypia **b**. The pattern of endometrial glandular dysplasia (EmGD) was shown in another field **c**. Higher magnification (original magnification *400) showed the presence of nuclear atypia in EmGD (inserted in the *left lower corner* of **c**). The cells in EmGD showed p53 over expression **d**, a relatively lower Ki67 index (approximately 40 %) **e**, and a moderate ER expression (++) **f**. The neoplastic cells in serous carcinoma and EIC (*arrows*) were diffusely positive for p53 **g**. (Original magnifications: A*50; B-G*200)

The preoperative endometrial curetting consisted of a small amount of specimen (measured 0.3 × 0.3 × 0.2 cm) with artifact crush. It showed a typical pattern of serous carcinoma including the papillary structure with a slender fibrous core and the highly atypical lining epithelial cells. In the resected uterus, only a small amount of serous carcinoma was found in 3 foci (each with a diameter of <0.5 mm) from 17 slides from the whole endometrium with partial myometrium. They were all located within the endometrium, and did not show evidence of myometrial invasion. Serous EIC and Endometrial glandular dysplasia (EmGD) was not found in the eutopic endometrium. Three small foci of serous neoplasia (2 mm-3 mm in diameters) were found from 2 of 12 slides from the region of adenomyosis. They were clearly separated from the basalis of the endometrium. Two lesions showed a minimal serous carcinoma and a serous EIC (Fig. 2a, 2g). The neoplastic cells showed significant atypia including nuclear pleomorphism and prominent nucleoli (Fig. 2b). The third lesion contained a predominant component of EmGD (Fig. 2c) and a minor serous EIC. All these lesions were surrounded by stromal cells. Lymphovascular invasion was occasionally found in the myometrium. Immunohistochemistry showed that both SC in the endometrial curetting and SC/serous EIC in the adenomyosis had the same immunostaining pattern (Table 4). The neoplastic cells were diffusely positive for p53 and Ki67, and negative for ER, PR and WT1 (Fig. 2h). The cells in EmGD showed an identical staining pattern except for a moderate ER expression and a relatively lower Ki67 index (approximately 40 %) (Fig. 2d-f).

#### Case 3

The cervical stump measured 5 × 5 × 4.5 cm. The mucosa looked a little bit rough, but no tumor was grossly seen in the cervical canal. A 3 × 1.5 × 1.5-cm mass was recognizable in the myometrium. The mass had 3 small cysts containing brownish, viscous fluid. The diameters of these cysts varied between 0.5-1.0 cm. These cysts were at least 7 mm from the endocervical surface. The left ovarian cyst showed the similar gross features and had a size of 4 × 3.5 × 3 cm. The resected rectum measured 5.5 cm long. The intestinal wall was partly sclerosing and hemorrhagic. The lumen became narrow, but had no macroscopic tumors or polyps. The cervical stump (*n* = 15) was extensively examined for microscopic assessment.

**Fig. 3:**
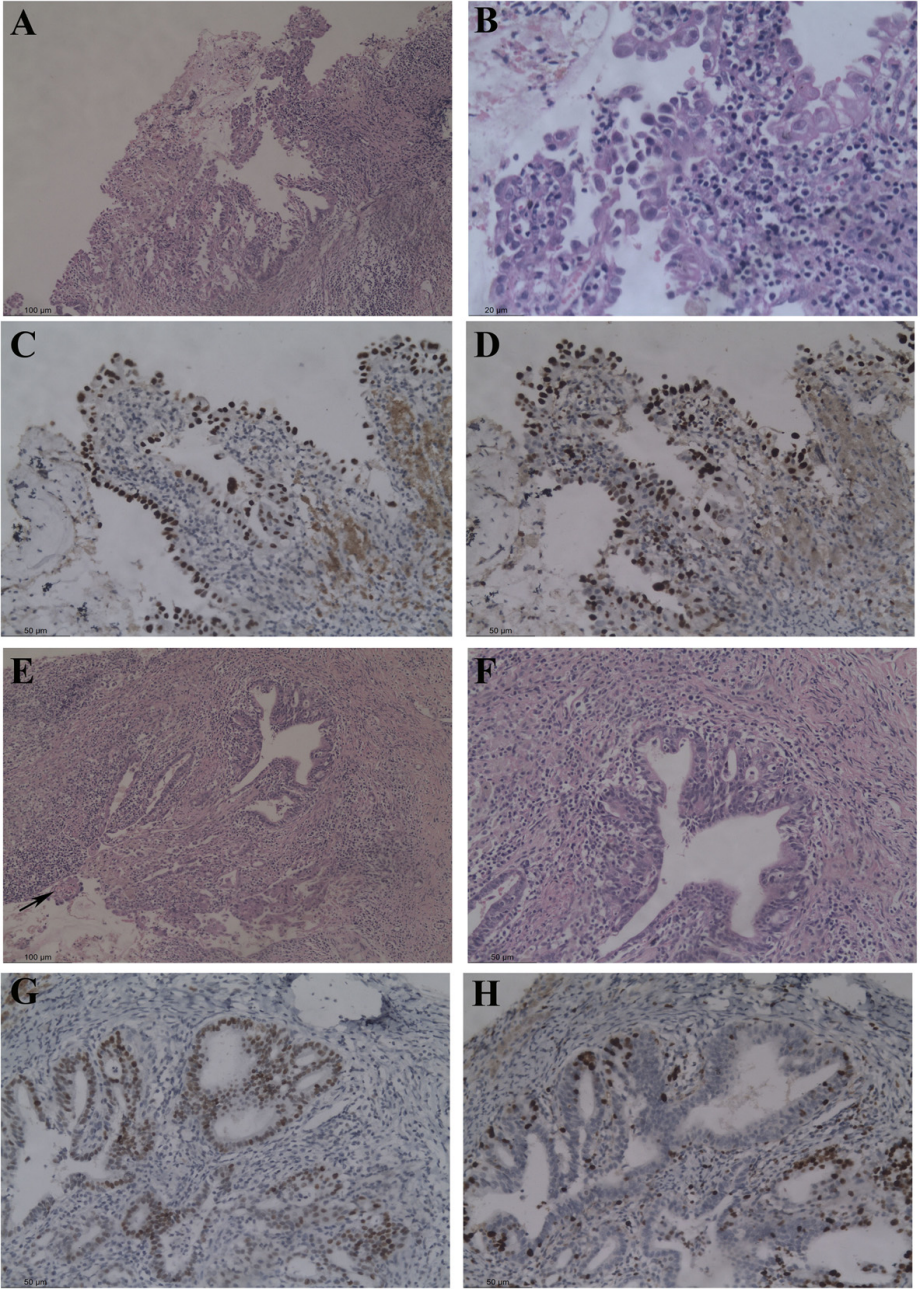
Serous EIC in adenomyotic cyst of the cervical stump (case 3). Serous EIC was present in the adenomyotic cysts by showing a slightly papillary contour with fibrous cores and the cells displayed hobnail cellular morphology and significant atypia **a**, **b**. The serous EIC showed p53 over expression **c** and a high Ki67 index **d**. A focus of EmGD and serous EIC (arrow) was seen in the adjacent area of one serous EIC lesion **e**, **f**. The EmGD lesion showed p53 over expression **g** and a relatively lower Ki67 index **h**. (Original magnifications: A, E*50; C, D*100; B, F-H*200)

The preoperative endocervical biopsy showed a predominantly solid pattern and a minor component of glandular structures in keeping with a poorly differentiated endometrioid carcinoma (grade 2–3). However, no residual tumors were found in the removed cervical stump. A 0.4-cm lesion of hemorrhage with abundant hemosiderin-containing macrophages was noted at the surface of the cervical mucosa. Three microscopic foci of serous EIC (each 1 mm in diameter) were noted in 2 of 3 adenomyotic cysts in the cervical stump. They had a slightly papillary contour with fibrous cores and the cells displayed hobnail cellular morphology and significant atypia (Fig. 3a, b). A focus of EmGD was seen in the adjacent area of one serous EIC lesion (Fig. 3e, f). Endometriosis was present in 4 slides from the surrounding cervical myometrium and parametrium. The left ovarian lesion was an endometriotic cyst. The rectal serosa and muscularis propria were significant for endometriosis. The immunostaining results of carcinoma in the endocervical biopsy and serous EIC in the adenomyotic cyst was shown in Table 4 (Fig. 3g, h). The EmGD lesion had an expression pattern identical to serous EIC except the lower Ki67 index (30 %) and patchy p16 staining (Fig. 3e, f).

## Discussion

Carcinoma arising from adenomyosis was initially reported more than one century ago. Sampson or Colman’s criteria for the diagnosis of carcinoma arising from adenomyosis were proposed early in 1950s [11, 12]. These includes: “1) the carcinoma must not be present in the normally situated endometrium or elsewhere in the pelvis; b) the carcinoma must be seen to arise from the epithelium within the adenomyosis rather than invasion from another source; and c) endometrial (adenomyotic) stromal cells must be seen to support a diagnosis of adenomyosis”. In fact, these criteria appear to be too stringent. In previous reports, 3 of 5 serous EICs and 1 of 4 carcinomas could not be classified as true tumors arising in adenomyosis if all 3 criteria should fully be satisfied [8, 9]. However, the authors provided compelling evidence to support that their cases should be considered as originating from adenomyosis.

In this study, we also understand that our cases do not meet the Sampson or Colman’s criteria completely, but we think that they are most likely from adenomyosis as we discuss below. The point most contradictory to the criteria in case 1 is the coexistence of uterine and ovarian carcinoma with the identical morphological and immunostaining features. The question arises that both carcinomas are primary or one is metastatic from the other. However, we believe that the ovarian lesion is unlikely to be primary according to the following findings. First, the ovarian carcinoma was grossly unapparent while the uterine carcinoma was obvious. The uterine tumor was deeply located in the myometrium. It had a relatively clear boundary and grossly looked like an adenomyoma (adenomyosis). Moreover, there was no tumor in the pelvis beyond the left ovary and uterus. In particular, serous carcinoma and its precursor lesions were not seen in the endometrium and cervix even after an extensive microscopic inspection on these sites. Second, the ovarian carcinoma was characterized by overwhelmingly glandular structures with severe cytological atypia, but no papillary formation was seen. The great disagreement of structure (G1) and nuclear grading (G3) is a classical diagnostic clue for endometrial serous carcinoma [13]. A diffuse glandular pattern is not a common feature for ovarian serous carcinoma. Moreover, no STIC and ovarian endometriosis was found in both fallopian tubes and ovaries. The frequency of STICs was 61 % in pelvic high grade serous carcinoma [14]. Endometriotic cyst can be an uncommon source of ovarian or pelvic serous carcinoma [2, 15, 16]. In contrast, the uterine carcinoma showed a pattern of neoplastic glands intermingled with smooth muscles, architecturally resembling an adenomyoma. Endometriosis was found in the tumor and the surrounding myometrium. Third, both uterine and ovarian cancers were totally negative for WT1 and ER, and had p53 over expression and a high Ki67 index. It was well documented that WT1 and ER expression was commonly present in ovarian serous carcinomas with a frequency of approximately 90 %, and very unusual in endometrial serous carcinoma [17–19]. Therefore, the WT1-ve and ER -ve pattern in both tumors of this case indicated that serous carcinoma in the uterine myometrium was most likely primary from adenomyosis, which had largely been replaced by tumor overgrowth. Accordingly, the ovarian carcinoma was secondary from the direct extension or lymphovascular spread of carcinoma in the uterine adenomyosis.

The presence of carcinoma in the endometrial cavity (case 2) and endocervical tube (case 3) may throw doubt on the origin of the endometriotic carcinoma from adenomyosis. We think that these cases can be explained by synchronous growth of endometrial and endometriotic carcinomas as reported previously [8]. In case 2, adenomyosis was diffuse and mostly was uninvolved by the serous cancer cells. The 3 involved foci of adenomyosis were separated clearly from the basalis of the endometrium while the small endometrial carcinoma was completely limited within the endometrium. In case 3, there was no residue tumor at the surface of the endocervix in the surgical specimens. The histotypes were different between the original endocervical biopsy and the lesions in the cervical adenomyosis. These lesions in the adenomyotic cyst were at least 7 mm from the endocervical surface. Moreover, all these microscopic lesions in the myometrium from case 2 and case 3 were clearly surrounded by benign stromal cells. They had foci of EmGD and serous EIC. Serous EIC is the established precursor lesion of endometrial serous carcinoma while EmGD is an emerging early lesion preceding EIC [20–22]. Kumar et al. [23] also emphasized that the presence of either a transition or continuity between the benign adenomyotic endometrial glands and the carcinomatous glands was essential to prove the adenomyotic origin of the carcinoma and to exclude the possibility of metastasis or invasion from elsewhere. The coexistence of EmGD and EIC in our cases indicates the interrelationships between the ectopic endometrium and serous carcinoma, thus makes metastasis from the eutopic endometrium most unlikely.

Two additional points in our cases deserved further discussion. Lymphovascular invasion is uncommon in EIC although EIC can spread in the peritoneum [24]. However, the distinction of extensive EIC and early invasive serous carcinoma has not been well established. According to the criteria suggested by Dr Wheeler DT, et al. [25], serous carcinoma in the endometrium and adenomyosis from case 2 may both represent for a minimally invasive serous carcinoma and can cause lymphovascular invasion. However, we think that the lymphovascular invasion is most likely associated with serous carcinoma from adenomyosis because it is present in the myometrium. Moreover, the endometrial serous carcinoma did not show evidence of myometrial invasion. In case 3, the presence of hobnail cell changes in EIC showed overlapping features with minute clear cell carcinoma or clear cell carcinoma in situ. However, such hobnail cellular changes can also be present in EIC [26]. HNF1β and Napsin A are typically expressed by clear cell carcinoma [27, 28], but they are completely negative in this case (data not shown). Taken the presence of EmGD together, our case is regarded as EIC most likely.

Two broad categories, type I and II, endometrial carcinomas have been well documented [29]. On the basis of these two categories, a “dualistic model” of endometrial carcinogenesis has been developed recently with the accumulative clinical, morphological and molecular evidence [30]. The prototype of type I carcinoma is endometrial carcinoma, which is associated with unopposed estrogen stimulus, and favorable prognosis. Serous carcinoma is the prototype of type II carcinoma. It is unrelated to the effect of unopposed estrogen. It invariably harbors p53 mutation and has poor prognosis. The type I carcinoma is much more common than type II carcinoma in the uterine endometrium. Carcinoma from ectopic endometrium is not very uncommon, and most in ovarian endometriosis. The ovarian carcinoma associated with endometriosis predominantly represents for endometrioid and clear cell types [4–6]. In the less than 50 well documented carcinomas arising from adenomyosis, only three were serous carcinoma or serous papillary carcinoma [7–10]. These data suggest that carcinomas arising from the ectopic endometrium including adenomyosis are predominantly type I over type II. Our report not only adds new cases to this rare carcinoma arising from adenomyosis, but also provides a characteristic immunostaining profile (PTEN+, ER-, membranous β-catenin+, p53 and p16 over expression, high Ki67 index) to uncover the distinct molecular alterations in type II carcinoma. Abushahin et al. [8] proved the presence of p53 mutation in 1 of 5 serous EICs arising in adenomyosis. That study also inferred that EmGD and serous EIC, arising in adenomyosis may be underdiagnosed because of their small foci in adenomyosis. In favor of their opinion, we observed that serous carcinomas in adenomyosis may also be under-recognized particularly when carcinoma occurred simultaneously in the endometrium or outside the uterus.

Carcinomas of the cervical stump after subtotal hysterectomy are not uncommon with an incidence of 0.1-3 %. Among these carcinomas, approximately 88 % were squamous cell carcinoma and the remaining were adenocarcinoma [31, 32]. However, we could not find any reports on adenocarcinoma or serous EIC arising in endometriosis (adenomyotic cyst) of the cervical stump in English literature to date. Case 3 demonstrated the presence of serous EIC in adenomyotic cyst in the cervical stump following the subtotal hysterectomy for adenomyosis. Gynecologists might take this rare complication into account in addition to the preservation of possible physiologic and sexual functions of the cervix when they decided to perform a proper surgery for women with diffuse uterine adenomyosis.

## Conclusions

In summary, our cases present a rare phenomenon that serous carcinoma and EIC can arise from ectopic endometrium including uterine adenomyosis and adenomyotic cyst in the cervical stump. Their small size as well as their inconspicuous histological features under scanning magnification may cause these lesions to be overlooked. Such cases may potentially become the unrecognizable source of extra-uterine or pelvic spread. These cases highlight the necessity of regular clinical follow up in patients with untreated uterine adenomyosis or subtotal hysterectomy for adenomyosis, and more meticulous pathological inspection on the resected uterine specimens. However, more studies are necessary to investigate the epidemiology and pathogenesis in serous carcinoma and EIC arising from ectopic endometrium, and to develop reliable approaches to delineate these cases from benign lesions preoperatively.

## Abbreviations

EIC, endometrial intraepithelial carcinoma; EmGD, endometrial glandular dysplasia; TAHBSO, total abdominal hysterectomy and bilateral salpingo-oophorectmy

## References

[1] Pearce CL, Templeman C, Rossing MA, Lee A, Near AM, Webb PM, Ovarian Cancer Association Consortium (2012). Association between endometriosis and risk of histological subtypes of ovarian cancer: a pooled analysis of case–control studies. Lancet Oncol.

[2] Boyraz G, Selcuk I, Yazıcıoğlu A, Tuncer ZS (2013). Ovarian carcinoma associated with endometriosis. Eur J Obstet Gynecol Reprod Biol.

[3] Acién P, Velasco I, Acién M, Capello C, Vela P (2015). Epithelial ovarian cancers and endometriosis. Gynecol Obstet Invest.

[4] Koike N, Tsunemi T, Uekuri C, Akasaka J, Ito F, Shigemitsu A, Kobayashi H (2013). Pathogenesis and malignant transformation of adenomyosis (review). Oncol Rep.

[5] Baba A, Yamazoe S, Dogru M, Ogawa M, Takamatsu K, Miyauchi J (2016). Clear cell adenocarcinoma arising from adenomyotic cyst: a case report and literature review. J Obstet Gynaecol Res.

[6] Kazandi M, Zeybek B, Terek MC, Zekioglu O, Ozdemir N, Oztekin K (2010). Grade 2 endometrioid adenocarcinoma arising from adenomyosis of the uterus: report of a case. Eur J Gynaecol Oncol.

[7] Izadi-Mood N, Samadi N, Sarmadi S, Eftekhar Z (2007). Papillary serous carcinoma arising from adenomyosis presenting as intramural leiomyoma. Arch Iran Med.

[8] Abushahin N, Zhang T, Chiang S, Zhang X, Hatch K, Zheng W (2011). Serous endometrial intraepithelial carcinoma arising in adenomyosis: a report of 5 cases. Int J Gynecol Pathol.

[9] Koshiyama M, Suzuki A, Ozawa M, Fujita K, Sakakibara A, Kawamura M (2002). Adenocarcinomas arising from uterine adenomyosis: a report of four cases. Int J Gynecol Pathol.

[10] Griffin M, Parai S, Ghatage P, Dimakulangan A (1996). Papillary serous carcinoma of the uterus arising in an adenomyoma. Acta Cytol.

[11] Sampson JA (1925). Endometrial carcinoma of the ovary arising in endometrial tissue of that organ. Arch Surg.

[12] Colman HI, Rosenthal AH (1959). Carcinoma developing in areas of adenomyosis. Obstet Gynecol.

[13] Garg K, Soslow RA (2012). Strategies for distinguishing low-grade endometrioid and serous carcinomas of endometrium. Adv Anat Pathol.

[14] Przybycin CG, Kurman RJ, Ronnett BM, Shih IM, Vang R (2010). Are all pelvic (nonuterine) serous carcinomas of tubal origin?. Am J Surg Pathol.

[15] Takeuchi K, Yamanaka Y, Hamana S, Ohara N, Maruo T (2004). Invasive adenocarcinoma arising from uterine adenomyosis involving the rectosigmoid colon. Int J Gynecol Cancer.

[16] Slomovitz BM, Soslow RA, Chang RC, Golub R, Kuo DY (2002). Serous adenocarcinoma of the inguinal region arising from endometriosis followed by a successful pregnancy. Gynecol Oncol.

[17] Bárcena C, Oliva E (2011). WT1 expression in the female genital tract. Adv Anat Pathol.

[18] Hirschowitz L, Ganesan R, McCluggage WG (2009). WT1, p53 and hormone receptor expression in uterine serous carcinoma. Histopathology.

[19] Zhang Y, Garcia-Buitrago MT, Koru-Sengul T, Schuman S, Ganjei-Azar P (2013). An immunohistochemical panel to distinguish ovarian from uterine serous papillary carcinomas. Int J Gynecol Pathol.

[20] Sherman ME, Bitterman P, Rosenshein NB, Delgado G, Kurman RJ (1992). Uterine serous carcinoma. A morphologically diverse neoplasm with unifying clinicopathologic features. Am J Surg Pathol.

[21] Zheng W, Xiang L, Fadare O, Kong B (2011). A proposed model for endometrial serous carcinogenesis. Am J Surg Pathol.

[22] Zheng W, Liang SX, Yu H, Rutherford T, Chambers SK, Schwartz PE (2004). Endometrial glandular dysplasia: a newly defined precursor lesion of uterine papillary serous carcinoma. Part I: morphologic features. Int J Surg Pathol.

[23] Kumar D, Anderson W (1958). Malignancy in endometriosis interna. J Obstet Gynaecol Br Emp.

[24] Soslow RA, Pirog E, Isacson C (2000). Endometrial intraepithelial carcinoma with associated peritoneal carcinomatosis. Am J Surg Pathol.

[25] Wheeler DT, Bell KA, Kurman RJ, Sherman ME (2000). Minimal uterine serous carcinoma: diagnosis and clinicopathologic correlation. Am J Surg Pathol.

[26] Ellenson LH, Ronnett BM, Kurman RJ, Kurman RJ, Ronnett BM, Ellenson LH (2011). Precursor lesions of endometrial carcinoma. Blaustein’s pathology of the female genital tract. 6^th^.

[27] Yamashita Y, Nagasaka T, Naiki-Ito A (2015). Napsin A is a specific marker for ovarian clear cell adenocarcinoma. Mod Pathol.

[28] Fadare O, Zhao C, Khabele D, Parkash V, Quick CM, Gwin K, Desouki MM (2015). Comparative analysis of Napsin A, alpha-methylacyl-coenzyme A racemase (AMACR, P504S), and hepatocyte nuclear factor 1 beta as diagnostic markers of ovarian clear cell carcinoma: an immunohistochemical study of 279 ovarian tumours. Pathology.

[29] Bokhman JV (1983). Two pathogenetic types of endometrial carcinoma. Gynecol Oncol.

[30] Lax SF, Kurman RJ (1997). A dualistic model for endometrial carcinogenesis based on immunohistochemical and molecular genetic analyses. Verh Dtsch Ges Pathol.

[31] Hellström AC, Hellman K, Pettersson BF, Andersson S (2011). Carcinoma of the cervical stump: fifty years of experience. Oncol Rep.

[32] Hellström AC, Sigurjonson T, Pettersson F (2001). Carcinoma of the cervical stump. The radiumhemmet series 1959–1987. Treatment and prognosis. Acta Obstet Gynecol Scand.

